# Numerical Study of Entropy Generation due to Coupled Laminar and Turbulent Mixed Convection and Thermal Radiation in an Enclosure Filled with a Semitransparent Medium

**DOI:** 10.1155/2014/761745

**Published:** 2014-03-20

**Authors:** M. Goodarzi, M. R. Safaei, Hakan F. Oztop, A. Karimipour, E. Sadeghinezhad, M. Dahari, S. N. Kazi, N. Jomhari

**Affiliations:** ^1^Department of Software Engineering, Faculty of Computer Science & Information Technology, University of Malaya, 50603 Kuala Lumpur, Malaysia; ^2^Department of Mechanical Engineering, Faculty of Engineering, University of Malaya, 50603 Kuala Lumpur, Malaysia; ^3^Department of Mechanical Engineering, Technology Faculty, Firat University, Elazig, Turkey; ^4^Department of Mechanical Engineering, Najafabad Branch, Islamic Azad University, Isfahan, Iran

## Abstract

The effect of radiation on laminar and turbulent mixed convection heat transfer of a semitransparent medium in a square enclosure was studied numerically using the Finite Volume Method. A structured mesh and the SIMPLE algorithm were utilized to model the governing equations. Turbulence and radiation were modeled with the RNG *k*-*ε* model and Discrete Ordinates (DO) model, respectively. For Richardson numbers ranging from 0.1 to 10, simulations were performed for Rayleigh numbers in laminar flow (10^4^) and turbulent flow (10^8^). The model predictions were validated against previous numerical studies and good agreement was observed. The simulated results indicate that for laminar and turbulent motion states, computing the radiation heat transfer significantly enhanced the Nusselt number (Nu) as well as the heat transfer coefficient. Higher Richardson numbers did not noticeably affect the average Nusselt number and corresponding heat transfer rate. Besides, as expected, the heat transfer rate for the turbulent flow regime surpassed that in the laminar regime. The simulations additionally demonstrated that for a constant Richardson number, computing the radiation heat transfer majorly affected the heat transfer structure in the enclosure; however, its impact on the fluid flow structure was negligible.

## 1. Introduction

The heat transfer phenomenon in which natural and forced convections occur simultaneously is known as mixed convection heat transfer. The mixed convection heat transfer is a fundamentally important heat transfer mechanism that takes place in many industrial and technological processes such as designing solar collectors, double-layer glass, buildings for thermal comfort, and cooling electronic parts.

Nonetheless, in applications related to large enclosures, the Rayleigh number is often very big, meaning that the nature of convection in the enclosure is completely turbulent. Owing to this state, the analysis of turbulent flows inside enclosures is still a challenging subject in fluid mechanics. The reason is that in experimental studies, measuring low speeds in the boundary layers of enclosures with the available probes or sensors is a difficult and daunting task. From a numerical perspective, although new methods including DES, LES, and DNS have achieved significant progress, it is still nearly impossible to completely analyze the stratification in the core of an enclosure. Nonlinearity and coupling of the governing equations make the calculations complicated and time consuming [1]. The complexity of calculating mixed convection has prompted researchers to study natural convection. Reference can be made to works accomplished by Braga and de Lemos [2], Kuznetsov and Sheremet [3], and Sheremet [4] among others.

In several instances, however, radiation heat transfer coexists with and has significant impact on the fluid structure. Due to its complexity though, its effect has unfortunately been overlooked in many research works [5–8].

The literature review demonstrates that no extensive studies have been done on the interaction between turbulent mixed convection heat transfer and radiation inside enclosures. Only a limited number of studies consider the effect of radiation on natural convection. As such, these researchers generally focus on air or other neutral gases as radiatively nonparticipating mediums owing to the simplicity of modeling radiation as well as the possibility to solve the governing equations and compute the average Nusselt number ($\text{N}\bar{\text{u}}$) of radiation and convection separately [9–11]. For semitransparent media like water, the convection and radiation governing equations are coupled with each other and therefore should be solved together. In this state, the total Nusselt number (Nu_total_) cannot be obtained by normally mathematically adding the radiative and convective Nusselt numbers.

The presented literature review indicates that the study of radiation as an effective thermal source is in its early stages. In particular, the effect that radiation has on convection heat transfer in the turbulent flow regime in semitransparent media is not entirely understood. The current study thus investigates the laminar and turbulent mixed convection heat transfer of water in a square enclosure in the presence of thermal radiation. The RNG *k*-*ε* turbulence model [12, 13] was applied for turbulent flow analysis. Model validation was accomplished by comparing the simulation results for laminar and turbulent flow regimes with the results found in the literature. The numerical results for streamlines, temperature, entropy and heat transfer in terms of average Nusselt number are presented.

## 2. The Governing Equations of Laminar and Turbulent Mixed Convection in Combination with Radiation

Continuity equation:

(1) $$\begin{array}{c} \frac{\partial u}{\partial x}+\frac{\partial v}{\partial y}=0. \end{array}$$

*X*  and *Y* Momentum Equations:

(2) ∂u∂t+u∂u∂x+v∂u∂y =−1ρ∂p∂x+∂∂x(v+vt)(2∂u∂x)+∂∂y(v+vt)(∂u∂y+∂v∂x),∂v∂t+u∂v∂x+v∂v∂y =−1ρ∂p∂y+gβ(T−Tm)+∂∂y(v+vt)(2∂v∂y)  +∂∂x(v+vt)(∂v∂x+∂u∂y).

Energy equation:

(3) ∂T∂t+u∂T∂x+v∂T∂y =∂∂x(vPr+vtσT)∂T∂x+∂∂y(vPr+vtσT)∂T∂y.

Turbulent Kinetic Energy Transport (*K*) equation:

(4) ∂k∂t+u∂k∂x+v∂k∂y =∂∂x(v+vtσk)∂k∂x+∂∂y(v+vtσk)∂k∂y+Pk+Gk−ε.

Dissipation of Turbulent Kinetic Energy Transport (*ε*) equation:

(5) ∂ε∂t+u∂ε∂x+v∂ε∂y =∂∂x(v+vtσε)∂ε∂x+∂∂y(v+vtσε)∂ε∂y+C1εkPk  +C2ε2k+C3εkGk−Rε.

The Eddy Viscosity from the Prandtl-Kolmogorov Relation is obtained by

(6) $$\begin{array}{c} v_{t}=C_{\mu }f_{\mu }\frac{k^{2}}{\varepsilon }. \end{array}$$

The Stress Production term, *P*_*k*_, can also be obtained by

(7) $$\begin{array}{c} P_{k}=v_{t}\left[2{\left(\frac{\partial u}{\partial x}\right)}^{2}+2{\left(\frac{\partial v}{\partial x}\right)}^{2}+{\left(\frac{\partial u}{\partial y}+\frac{\partial v}{\partial y}\right)}^{2}\right]. \end{array}$$

The Buoyancy Term, *G*_*k*_, can be expressed as follows:

(8) $$\begin{array}{c} G_{k}=-g\beta \frac{v_{t}}{\sigma_{t}}\frac{\partial T}{\partial y}. \end{array}$$

For term *R*_*ε*_ in equation *ε* we have

(9) $$\begin{array}{c} R_{\varepsilon }=\frac{C_{\mu }\rho \eta^{3}\left(1-\left(\eta /\eta_{0}\right)\right)}{1+\beta \eta^{3}}\frac{\varepsilon^{2}}{k},\\ \eta =\frac{Sk}{\varepsilon }, \end{array}$$

where the turbulence model coefficients are as follows:

(10) $$\begin{array}{c} C_{\mu }=0.0845,\;\;\sigma_{k}=1,\;\;\sigma_{\varepsilon }=1.3,\\ C_{1}=1.42,\;\;C_{2}=1.68,\;\;\eta_{0}=4.38,\\ \beta =0.012,\;\;K=0.41. \end{array}$$

The Discrete Ordinates (DO) radiation model for spectral intensity is [12, 15]:

(11) ∇·(Iλ(r→,s→)s→)+(aλ+σs)Iλ(r→,s→) =aλn2Ibλ+σs4π∫04πIλ(r→,s→′)ϕ(s→,s→′)dΩ′,

where *λ* is the wavelength, *a*_*λ*_ is the spectral absorption coefficient, and *I*_*bλ*_ is the black body intensity provided by the Planck function.

The total intensity $\left(I\left(\vec{r},\vec{s}\right)\right)$ in direction $\vec{s}$ and at position $\vec{r}$ is calculated by

(12) $$\begin{array}{c} I\left(\vec{r},\vec{s}\right)=\sum_{k}I_{\lambda_{k}}\left(\vec{r},\vec{s}\right)\Delta \lambda_{k}, \end{array}$$

where the summation is over the wavelength bands.

Entropy Generation [16]:

(13) Sgen=kT2[(∂T∂x)2+(∂T∂y)2]+μT{2[(∂υ∂x)2+(∂υ∂y)2]+(∂υ∂y+∂υ∂x)2}.

## 3. Boundary Conditions

**Figure 1:**
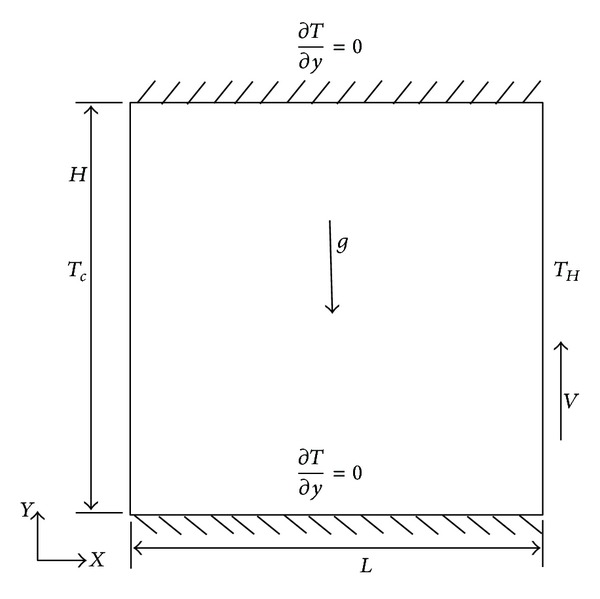
Schematic of analyzed configuration.

Figure 1 illustrates a schematic of the configuration analyzed in the present study along with the boundary conditions.

The specific boundary conditions for the present study are:

(14) $$\begin{array}{c} T=T_{c},\;u=v=0,\;0<y<1,\;x=0,\\ T=T_{h},\;v=v_{\text{lid}},\;u=0,\;0<y<1,\;x=1,\\ \frac{\partial T}{\partial y}=0,\;u=v=0,\;0<x<1,\;y=0,\\ \frac{\partial T}{\partial y}=0,\;u=v=0,\;0<x<1,\;y=1. \end{array}$$

### 3.1. Wall Function Modeling

The standard wall function described by Launder and Spalding [17] and used in Abedini et al. [18] and Goodarzi et al. [19] is a semiempirical formula based on the established properties of turbulence in the inertial sub-layer near a wall. In this approach, the velocity at the first grid is given as follows:

(15) $$\begin{array}{c} U^{+}=2.389\ln\left(9.793Y^{+}\right), \end{array}$$

where

(16) $$\begin{array}{c} U^{+}=\frac{U_{P}C_{\mu }^{0.25}K_{P}^{0.5}}{\tau_{w}/\rho },\\ y^{+}=\frac{\rho Y_{P}C_{\mu }^{0.25}K_{P}^{0.5}}{\mu }. \end{array}$$

The logarithmic law for the mean velocity is valid for the range 11 ≤ *y* ^+^ ≤ 300. When the meshes are in such a way that *y* ^+^ ≤ 11 at the wall-adjacent cells, in the viscous sublayer, the linear velocity profile holds. That is,

(17) $$\begin{array}{c} U^{+}=Y^{+}. \end{array}$$

For the temperature boundary conditions:

(18) T+=ρCP(TW−TP)Cμ0.25KP0.5q˙={Pr Y+(Y+<YT+)0.85[2.389ln⁡(9.793Y+)+ζ](Y+>YT+),

where

(19) $$\begin{array}{c} \zeta =9.24\left[{\left(\frac{\text{Pr}}{0.85}\right)}^{0.75}-1\right]\left[1+0.28e^{-0.007\text{Pr}/0.85}\right]. \end{array}$$

For the turbulence *K*-*ε* model, the boundary conditions for turbulent kinetic energy are given as follows:

(20) $$\begin{array}{c} \frac{\partial K}{\partial Y}=0. \end{array}$$

The corresponding turbulence kinetic energy production term is given by

(21) $$\begin{array}{c} P_{k}\approx \tau_{w}\frac{\partial u}{\partial y}=\tau_{w}\frac{\tau_{w}}{0.4187\rho C_{\mu }^{0.25}Y_{p}K_{P}^{0.5}}. \end{array}$$

At the wall-adjacent cells, the *ε* equation is not solved. But instead, *ε*_*P*_ is evaluated as [19]:

(22) $$\begin{array}{c} \varepsilon_{P}=\frac{C_{\mu }^{0.75}K_{p}^{1.5}}{kY_{p}}. \end{array}$$

## 4. Numerical Method

In order to solve the governing equations, the FLUENT commercial code based on the finite volume method was used and is described in detail in [20]. The finite volume method is a specific case of the weighting residual method, where the computational field is divided into finite control volumes as each node corresponds to a control volume. The differential equation is subsequently integrated over each finite volume [21, 22].

The Second Order Upwind scheme was engaged to discretize the convective and diffusive terms, while the SIMPLE algorithm [23, 24] was selected for the pressure-velocity coupling. The calculation was considered converged when the residuals for all equations dropped below 10^−7^. Such criteria for the equations guarantee highly precise solutions [25].

## 5. Numerical Procedure Validation

### 5.1. Laminar Mixed Convection Validation

For the purpose of validating the laminar mixed convection flow part of the analysis, the problem described by Sharif [14] was solved and the present simulation results were compared with it. In [14], the laminar mixed convection heat transfer of water in a lid-driven cavity with an aspect ratio of 0.1, cooled from the bottom and heated from the top movable wall, was studied via the finite volume method. Calculations were done for 0.1 ≤ Ri ≤ 10, while the Reynolds number was kept fixed at *Re* = 408.21. The computed average Nusselt number in Ri = 1 was contrasted with the work of Sharif [14] in Table 1(a). The table illustrates excellent agreement between the present simulation results and those in Sharif [14]. Therefore, the current numerical procedure can be applied with confidence in the simulation of laminar mixed convection flows.

### 5.2. Turbulent Convection and Radiation Heat Transfer Validation

The present numerical procedure for solving laminar and turbulent convection conjugating radiation was verified against the existing results of Xamán et al. [10]. In that work, laminar and turbulent natural convection combined with surface radiation and conduction heat transfer in a square cavity filled with air was analyzed numerically by finite volume method. Calculations were done for 10^3^ ≤ Ra ≤ 10^12^, Δ*T* = 14°C and surface solar radiation 750 W m^−2^. For Ra = 10^4^ (laminar regime) and 10^12^ (turbulent regime), the average Nusselt number is shown in Table 1(b) and compared with the results of Xamán et al. [10]. Table 1(b) demonstrates reasonable concord between the outcomes of the present work and those of Xamán et al. [10]. The small discrepancies seen in this table may be due to having ignored the walls' conduction in the present study.

### 5.3. Grid Independence

Structured nonuniform grid distributions were applied to discretize the computation domain. The significance of the temperature and velocity gradients near the walls caused the grid to be more refined there. Various grid distribution types were tested to ensure the results were grid independent. The grid independence for each turbulence model and different Ri was tested separately. Tables 2(a) and 2(b) are two examples indicative of several tests carried out in the grid study.

## 6. Results and Discussion

This study was meant to analyze the effect of radiation on flow-induced buoyancy in a square enclosure (*H* = *L*). It was assumed that the horizontal walls of the enclosure are adiabatic; the right moving wall is *T*_1_ = 323 K and the left wall is *T*_2_ = 283 K with 1000 W/m^2^ irradiation in both walls. The enclosure walls are made of iron and are supposed to be opaque. Fluid density was computed by applying the Boussinesq approximation [9, 10]. The other thermophysical and optical properties of the fluid were assumed to be constants. In order to achieve a higher Ra, the dimensions of the enclosure were augmented.

Table 3 describes the average Nusselt number $\left(\text{N}\overset{-}{\text{u}}\right)$ values inside the enclosure for different Richardson numbers in the laminar and turbulent states of motion. The table clearly shows that for laminar and turbulent regimes, the average Nusselt number and therefore heat transfer rate inside the enclosure increased by over 20% and 35%, respectively when computing the radiation heat transfer in the calculation. It is also clear from this table that due to the great influence that radiation has on the nature of flow, the average Nusselt number remained nearly fixed for various Richardson numbers. In the other words, the effect of natural, mixed, or forced convection heat transfer inside the enclosure can totally disappear when the effect of radiation is considered in the calculation. Furthermore, the extra mixing in the turbulent flow regime leads to a higher heat transfer rate than in the laminar regime.

Table 4 illustrates the effect of radiation on the maximum values of stream function. At a quick glance, the effect of thermal radiation on fluid structure is obviously not remarkable. There is only less than 1% difference between the maximum values of stream functions in the presence of thermal radiation, meaning that the effect of radiation on the structure of fluid flow can be ignored due to the high cost of radiation computing.

**Figure 2:**
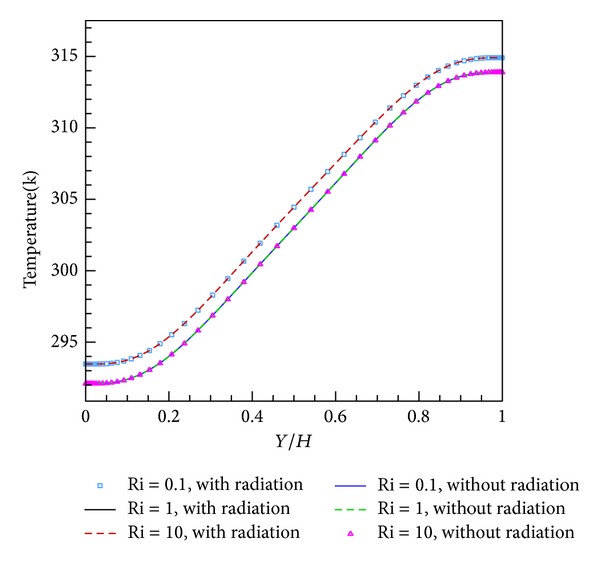
Temperature representation in mid-length for different Richardson numbers in laminar flow regime.

**Figure 3:**
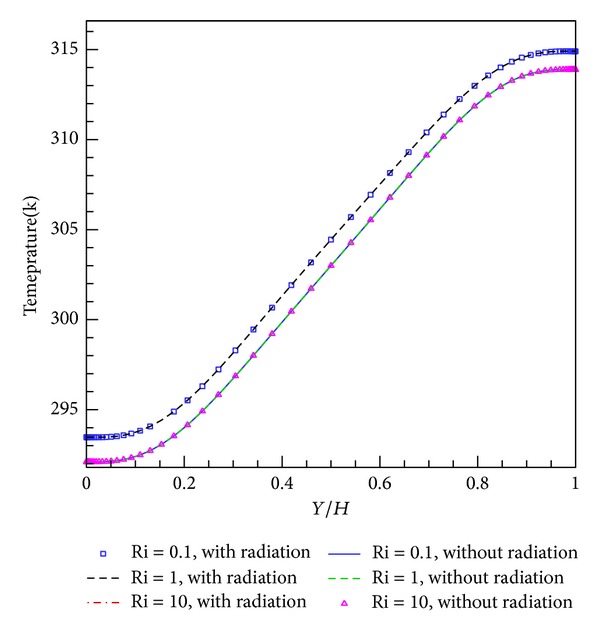
Temperature representation in mid-length for different Richardson numbers in turbulent flow regime.

For the laminar and turbulent flow regimes, Figures 2 and 3 illustrate the temperature diagrams at *X*/*L* = 0.5 inside the enclosure for different Richardson numbers. For both cases, the temperature diagram has a linear trend inside the height of the enclosure but for 0 ≤ *Y*/*H* ≤ 0.2 and 0.8 ≤ *Y*/*H* ≤ 1, one concavity and one convexity reduce the discipline of the temperature diagram. However, as seen in the figures, the influence of radiation on temperature is outstanding. In this state, the temperature difference range is more compact than with pure convection. The difference is 1.5 K near the upper wall and 1.3 K near the lower wall.

**Figure 4:**
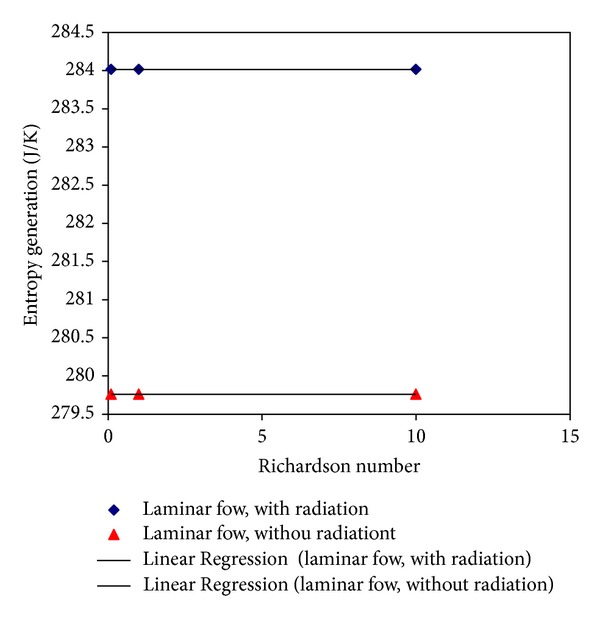
Average of entropy representation for different Richardson numbers in laminar flow regime.

**Figure 5:**
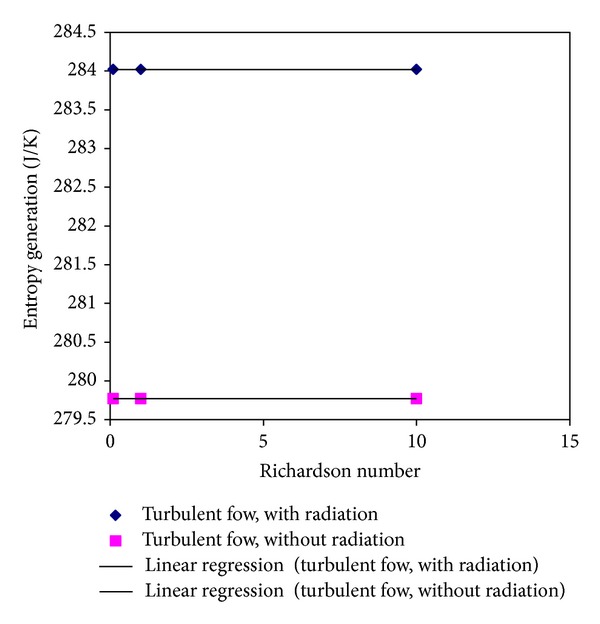
Average of entropy representation for different Richardson numbers in turbulent flow regime.

Figures 4 and 5 show the average of entropy diagrams for the laminar and turbulent regimes. It is evident that the values of entropy vaguely increase (roughly 4.3 J K^−1^) by computing radiation in the calculation. However, with linear regression, the correlations between the Richardson numbers and entropy are obtained as follows:

(23) Smax⁡=−7  ×  10−5Ri+284.02For  Laminar  Flow  and  the  presence  of  radiation,Smax⁡=−6  ×  10−5Ri+279.76For  Laminar  Flow  and  without  radiation,Smax⁡=−9  ×  10−6Ri+284.02For  Turbulent  Flow  and  the  presence  of  radiation,Smax⁡=−8  ×  10−6Ri+279.76For  Turbulent  Flow  and  without  radiation.

## 7. Conclusion

The present study investigated the combination of radiation with mixed convection heat transfer and fluid flow inside a square enclosure with a right hot moving wall. The main dimensionless parameter here was the Richardson number which varied from 0.1 to 10. The Rayleigh numbers were fixed at 10^4^ for laminar flow and 10^8^ for turbulent flow. The flow and temperature fields as well as various parameters like entropy and average Nusselt number were evaluated.

The study has led to the following conclusions:

- For both laminar and turbulent regimes, the heat transfer rate is enhanced by computing the effect of radiation.
- Due to additional mixing in the turbulent flow regime, the heat transfer rate is generally higher than in the laminar regime.
- For both laminar and turbulent regimes, in the presence of radiation heat transfer, the average Nusselt number is almost fixed for different Richardson numbers.
- At low and moderate temperature differences, the effect of radiation heat transfer on the structure of fluid flow is insignificant.

## Figures and Tables

**Table:** (a)

Ri, surface	$\text{N}\overset{-}{\text{u}}$ [14]	$\text{N}\overset{-}{\text{u}}$ (present study)
1, cold wall	3.27	3.273
1, hot wall	3.58	3.588

**Table:** (b)

Ra	$\text{N}\overset{-}{\text{u}}$ with radiation [10]	$\text{N}\overset{-}{\text{u}}$ without radiation [10]	$\text{N}\overset{-}{\text{u}}$ with radiation (present study)	$\text{N}\overset{-}{\text{u}}$ without radiation (present study)
10^4^	2.55	1.83	2.48	1.77
10^12^	972.51	591.27	969.96	589.02

**Table:** (a)

Number of grids	50 × 50	100 × 100	200 × 200
Average Nusselt number for Ri = 0.1	0.771516	0.771516	0.771512
Average Nusselt number for Ri = 1	0.7715094	0.7715094	0.7715082
Average Nusselt number for Ri = 10	0.7715067	0.7715067	0.7715053

**Table:** (b)

Number of grids (Ri = 0.1)	200 × 200	250 × 250	300 × 300
Average Nusselt number for Ri = 0.1	26.0878274	26.0878260	26.0878248
Number of grids (Ri = 1)	220 × 220	250 × 250	300 × 300
Average Nusselt number for Ri = 1	26.0839758	26.0839740	26.0839728
Number of grids (Ri = 10)	250 × 250	300 × 300	400 × 400
Average Nusselt number for Ri = 10	26.045232	26.045226	26.045223

**Table 3:** The effect of radiation on the average Nusselt number ($\text{N}\overset{-}{\text{u}}$).

Ra	$\text{N}\overset{-}{\text{u}}$ with radiation			$\text{N}\overset{-}{\text{u}}$ without radiation		
	Ri = 0.1	Ri = 1	Ri = 10	Ri = 0.1	Ri = 1	Ri = 10
10^4^ (Laminar Regime)	0.771514	0.771509	0.771506	0.642928	0.642925	0.642922
10^8^ (Turbulent Regime)	26.087826	26.083974	26.045226	19.295729	19.292880	19.292760

**Table 4:** The effect of radiation on the maximum value of stream function.

Ra	Ψ with radiation			Ψ without radiation		
	Ri = 0.1	Ri = 1	Ri = 10	Ri = 0.1	Ri = 1	Ri = 10
10^4^	0.0007711	0.0007710	0.0007710	0.0007682	0.0007678	0.0007672
10^8^	0.0007709	0.0007708	0.0007708	0.0007679	0.0007673	0.0007668

## Nomenclature

*P*: Pressure (N m^−2^)
*T*: Temperature (K)
*K*: Turbulence kinetic energy (m^2^ s^−2^)
*S*: Entropy (J K^−1^)
*t*: Time (s)
*u*, *v*: Velocities components in *X* and *Y* directions (m s^−1^)
*x*, *y*: Cartesian coordinates (m)
*h*: Heat transfer coefficient (W m^−2^ K^−1^)
*k*: Thermal conductivity (W m^−1^ K^−1^)
$\vec{g}$: Gravitational acceleration (m s^−2^)
*C*_*p*_: Specific heat capacity (J kg^−1^ K^−1^)
*K*_*t*_: Turbulent thermal conductivity (W m^−1^ K^−1^)
*I*_*bλ*_: Black body intensity (W m^−2^)
${\vec{a}}_{\lambda }$: Spectral absorption coefficient (m^−1^)
Nu: Nusselt number
Re: Reynolds number
Pr: Prandtl number (*υα* ^−1^)
Gr: Grashof number (*gβ*Δ*TL* ^3^ *υ* ^−2^)
Ra: Rayleigh number (Gr Pr)
Ri: Richardson number (Gr *Re* ^−2^).

### Greek Symbols

*μ*: Dynamic viscosity (Pa s)
*ρ*: Density (Kg m^−3^)
*β*: Thermal expansion coefficient (K^−1^)
*υ*: Kinematics viscosity (m^2^ s^−1^)
*ε*: Dissipation rate of turbulent kinetic energy (m^2^ s^−3^)
*α*: Thermal diffusivity (m^2^ s^−1^)
*υ*_*t*_: Turbulence eddy viscosity (m^2^ s^−1^)
*σ*_*T*_: Turbulent thermal diffusivity (m^2^ s^−1^)
*λ*: Wavelength (*μ*m).

### Subscripts

0: Inlet conditions.
